# Induction of CNS α-synuclein pathology by fibrillar and non-amyloidogenic recombinant α-synuclein

**DOI:** 10.1186/2051-5960-1-38

**Published:** 2013-07-17

**Authors:** Amanda N Sacino, Mieu Brooks, Nicholas H McGarvey, Alex B McKinney, Michael A Thomas, Yona Levites, Yong Ran, Todd E Golde, Benoit I Giasson

**Affiliations:** 1 Center for Translational Research in Neurodegenerative Disease, Department of Neuroscience, BMS Building J-483, University of Florida College of Medicine, 1275 Center Drive, PO Box 100159, Gainesville, FL 32610, USA

**Keywords:** Amyloid, Neonatal, Parkinson’s disease, Pathology, α-Synuclein, Transgenic mice

## Abstract

**Background:**

α-Synuclein (αS) is the major component of several types of brain inclusions including Lewy bodies, a hallmark of Parkinson’s disease. Aberrant aggregation of αS also is associated with cellular demise in multiple neurologic disorders collectively referred to as synucleinopathies. Recent studies demonstrate the induction of αS pathology by a single intracerebral injection of exogenous amyloidogenic αS in adult non-transgenic and transgenic mice expressing human αS. To further investigate the mechanism of pathology induction and evaluate an experimental paradigm with potential for higher throughput, we performed similar studies in neonatal mice injected with αS.

**Results:**

In non-transgenic mice, we observed limited induction of neuronal αS inclusions predominantly 8 months after brain injection of aggregated, amyloidogenic human αS. More robust inclusion pathology was induced in transgenic mice expressing wild-type human αS (line M20), and inclusion pathology was observed at earlier time points. Injection of a non-amyloidogenic (Δ71-82) deletion protein of αS was also able to induce similar pathology in a subset of M20 transgenic mice. M20 transgenic mice injected with amyloidogenic or non-amyloidogenic αS demonstrated a delayed and robust induction of brain neuroinflammation that occurs in mice with or without αS pathological inclusions implicating this mechanism in aggregate formation.

**Conclusions:**

The finding that a non-amyloidogenic Δ71-82 αS can induce pathology calls into question the simple interpretation that exogenous αS catalyzes aggregation and spread of intracellular αS pathology solely through a nucleation dependent conformational templating mechanism. These results indicate that several mechanisms may act synergistically or independently to promote the spread of αS pathology.

## Background

A characteristic of Parkinson’s disease, the most common neurodegenerative movement disorder, is the presence of intraneuronal Lewy bodies (LBs) in neurons. These inclusions are formed from the amyloidogenic aggregation of the normally soluble presynaptic protein α-synuclein (αS). αS brain inclusions also are present in a spectrum of neurodegenerative disorders known as α-synucleinopathies [1-3]. A direct causal role for αS in neurodegeneration is supported by missense mutations or increased copy number of the αS gene (*SNCA*) in some patients with Parkinson’s disease and the related disorder dementia with Lewy bodies [4-11]. Despite a large number of experimental studies, the precise mechanism(s) of αS toxicity is still not resolved, although multiple lines of evidence support the hypothesis that αS aggregation is linked to cellular demise [1,12].

α-Synucleinopathies are progressive diseases and in recent years there have been increasing efforts to identify the mechanisms involved in intracerebral spread of pathology, as it is reasoned that therapies that could slow or halt pathology spread would likely be disease modifying. Recently, several experimental and pathological studies have suggested that spreading of αS pathology might occur via a seeded conformational-templating protein aggregation mechanism. For example, LB formation was observed in fetal dopaminergic neurons of a subset of PD patients that received striatal transplants as an attempted therapeutic intervention [13-15]. A seeding mechanism would also generally be consistent with the proposed Braak staging of disease that appears to follow neuroanatomical pathways [16]. Experimentally it was reported that the intracerebral injection of extracts from moribund A53T human αS transgenic (Tg) mice (line M83) that develop a late onset severe motor phenotype associated with widespread formation of neuronal αS inclusions into younger healthy M83 Tg mice could induce these cellular and phenotypic pathologies [17-19]. Furthermore, brain injection of pre-formed recombinant αS fibrils into M83 Tg mice can also induce αS pathology within brain regions that are distant from the injection site [18], suggesting that these αS species can initiate and perhaps lead to transmission of αS pathology. Induction of brain αS pathology was also reported in non-Tg (nTg) mice following intrastriatal injection of murine fibrillar αS [20]. More recently it was reported that the injection of either preformed human or mouse αS fibrils in the substantia nigra of nTg mice could also induce neuronal αS pathology, but this pathology could only be observed 3 months or more after exposure [21]. Collectively these studies along with numerous *in vitro* and culture studies support the concept of αS pathology spread within the brain via a conformational templating mechanism. However, this mechanism of pathology induction remains to be formally proven *in vivo*, as other possible mechanisms could contribute to αS inclusion pathology induction including disruption of proteostasis and innate immune activation [22-25].

To further elucidate the mechanisms associated with the induction of intraneuronal αS inclusion pathology resulting from exogenous αS challenge and to evaluate a potentially higher throughput experimental paradigm, we injected amyloidogenic and non-amyloidogenic forms of αS into the brain of neonatal nTg and M20 Tg mice expressing wild-type human αS. Neonatal injection is a significantly easier and faster surgical procedure than stereotactic injection in the adult brain, mainly because cryo-anesthesia can be utilized and the skull is still soft and flexible. These studies reveal that neonatal cerebral injection of amyloidogenic αS results in limited neuronal αS inclusions in nTg mice that are observed predominantly 8 months after injection. Similar studies in M20 Tg mice also revealed a lag time in the formation of detectable αS pathology, but pathology was more widespread throughout the neuroaxis and was induced by the injection of both amyloidogenic and non-amyloidogenic forms of αS.

## Material and methods

### Antibodies

pSer129 is a mouse monoclonal antibody specific to αS phosphorylated at Ser129 [26]. Syn211 and LB509 are mouse monoclonal antibodies specific for human αS [27,28]. SNL-1 is a rabbit polyclonal antibody raised against a synthetic peptide corresponding to amino acids 104–119 of αS and specifically reacts with both murine and human αS [27]. SNL-4 is a rabbit polyclonal antibody raised against a synthetic peptide corresponding to amino acids 2–12 of αS [27]. Syn506 is a conformational anti-αS mouse monoclonal antibody that preferentially detects αS in pathological inclusions [29,30]. Anti-p62 (SQSTM1; Proteintech; Chicago, IL), anti-glial fibrillary acidic protein (GFAP; Promega; Madison, WI), and anti-ionized calcium-binding adaptor molecule 1 (IBA-1; DAKO; Glostrio, Denmark) are rabbit polyclonal antibodies. An anti-glyceraldehyde-3-phosphate dehydrogenase (GAPDH) mouse monoclonal antibody was obtained from Biodesign (Memphis, TN).

### nTg mice and M20 αS Tg mice

All procedures were performed according to the NIH Guide for the Care and Use of Experimental Animals and were approved by the University of Florida Institutional Animal Care and Use Committee. BL6C3HF1 mice (Charles River Laboratories International Inc, Wilmington, MA) have the same strain background as αS Tg mice (line M20) and were used as nTg mice. The M20 Tg mice express human wild-type αS under the control of the mouse PrP promoter and these mice do not develop any intrinsic phenotype or αS pathology [31,32]. Hemizygous M20 Tg male mice were mated with female BL6C3HF1 mice and genotyped by PCR, but also confirmed by immunohistochemical (IHC) staining of mouse brain section with anti-human αS antibody Syn211. All animals were housed 3 to 5 to a cage and maintained on ad libitum food and water with a 12 h light/dark cycle.

### Brain αS injection into neonatal mice

**Figure 1:**
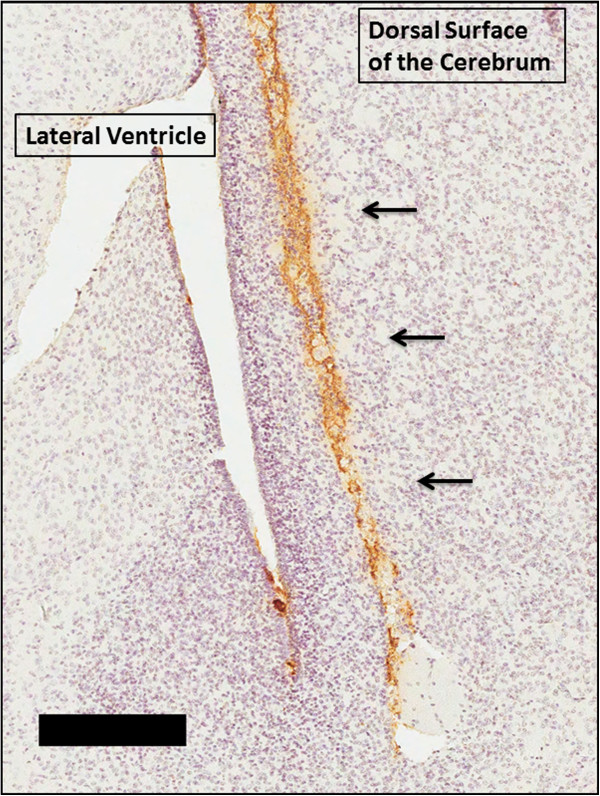
**Detection of injected human αS in the needle track 2 days post neonatal injection.** IHC staining with human αS specific antibody LB509 2 days after neonatal injection of 25 μg 21–140 human αS fibrils in nTg mice. Staining shows the presence of human αS in the brain injection tract adjacent to the lateral ventricle in the cerebrum (black arrows). The tissue section was counterstained with hematoxylin. Scale bar = 250 μm.

Bilateral neonatal (P0) injections of αS proteins were performed by inserting the needle about 0.5 cm deep into the brain just lateral to the lateral ventricles in the cerebrum (see Figure 1) using cryo-anesthesia as described previously [33]. In brief, P0 pups were cryo-anesthetized on ice for up to 5 minutes. Each pup received bilateral injections of αS proteins. Injections were made using a 10 μL Hamilton syringe with a 30-gauge needle. Different syringes were used for each type of protein to prevent any contamination. Post-injection, the pups were placed on a heating pad for recovery before being returned to their home cage.

### Expression and purification of recombinant αS proteins

The pRK172 cDNA constructions expressing full-length human αS, human αS with amino acid 71–82 deletion (Δ71-82), and N-terminal truncated 21–140 αS (with a Met codon added before amino acid 21) were previously described [34,35]. αS proteins were expressed in E. coli BL21 (DE3) and purified to homogeneity by size exclusion (Superdex 200 gel filtration) and ion exchanged (Mono Q) chromatographies as previously described [34,36].

### Fibril preparation of recombinant αS for mouse brain injection

21–140 αS protein was assembled into filaments by incubation at 37°C at 5 mg/ml in sterile phosphate buffered saline (PBS, Invitrogen) with continuous shaking at 1050 rpm (Thermomixer R, Eppendorf, Westbury, NY). αS amyloid fibril assembly was monitored as previously described with K114 fluorometry [35,37]. αS fibrils were diluted in sterile PBS and treated by water bath sonication for 2 hours. These fibrils were tested for induction of intracellular amyloid inclusion formation as previously described [38,39].

### Immunohistochemical analysis

Mice were sacrificed with CO_2_ euthanization and perfused with PBS/heparin, followed by perfusion with either 70% ethanol/150 mM NaCl or PBS buffered formalin. The brain and spinal cord were then removed and fixed for at least 24 hours in the respective fixatives used for perfusion. As previously described, tissues were dehydrated at room temperature through a series of ethanol solutions, followed by xylene and then were infiltrated with paraffin at 60°C [40]. The tissues were then embedded into paraffin blocks, which were cut into 7 μm sections. Immunostaining of the sections was performed using previously described methods [40] with the avidin-biotin complex (ABC) system (Vectastain ABC Elite Kit, Vector Laboratories, Burlingame, CA), and with immunocomplex visualization via chromogen 3, 3’-diaminobenzidine. Sections were counterstained with hematoxylin. All slides were scanned using an Aperio ScanScope CS (40× magnification; Aperio Technologies Inc., Vista, CA), and images of representative areas of αS pathology were taken using the ImageScopeTM software (40× magnification; Aperio Technologies Inc.).

### Double-labeling immunofluorescence analysis of mouse brain tissue

Paraffin-embedded tissue sections were deparaffinized and hydrated through a series of graded ethanol solutions followed by 0.1 M Tris, pH 7.6. The sections were blocked with 5% dry milk/0.1 M Tris, pH 7.6, and were incubated simultaneously with combinations of primary antibodies diluted in 5% dry milk/0.1 M Tris, pH 7.6. After extensive washing, sections were incubated with secondary antibodies conjugated to Alexa 594 or Alexa 488 (Invitrogen; Eugene, OR). Sections were post-fixed with formalin, incubated with Sudan Black, and stained with 5 μg/ml 4’, 6-diamindino-2-phenylindole (DAPI). The sections were coverslipped with Fluoromount-G (SouthernBiotech, Birmingham, AL) and visualized using an Olympus BX51 microscope mounted with a DP71 Olympus digital camera to capture images.

### Immunoblotting analysis

Mouse brains were lysed in 2% SDS/50 mM Tris pH 7.5 by sonication and heated to 100°C for 10 minutes. Protein concentration was quantified using the bicinchoninic acid (BCA) assay and bovine serum albumin as a standard (Pierce Biotechnology; Rockford, IL). 15 μg of total protein was resolved by SDS-PAGE on 13% polyacrylamide gels, followed by electrophoretic transfer onto nitrocellulose membranes. Membranes were blocked in Tris buffered saline (TBS) with 5% dry milk, and incubated with primary antibodies which were followed by either goat anti-mouse conjugated horseradish peroxidase (HRP) (Amersham Biosciences; Piscataway, NJ) or goat anti-rabbit HRP (Cell Signaling Technology; Danvers, MA). Protein bands were detected using chemiluminescent reagent (NEN; Boston, MA) and a FluorChem E and M Imager (Proteinsimple; San Jose, California).

### MALDI-TOF mass spectrometry of full-length αS and Δ71-82 αS

Recombinant human full-length αS and Δ71-82 αS (~4 mM) were diluted to 10 μM with 0.1% TFA (trifluoroacetic acid) solution. 1 μl diluted sample was mixed with 1 μl saturated ACCA (α-cyano-4-hydroxycinnamic acid) solution (acetonitrile: methanol = 3:2). 1 μl sample mixture was loaded to ACCA pretreated MSP 96 target (Bruker Daltonics Inc.; Billerica, MA). The samples were analyzed with a Bruker Microflex (Bruker Daltonics Inc.; Billerica, MA) mass spectrometer in linear positive model. Spectra were calibrated with Bruker protein calibrate standard.

## Results

**Figure 2:**
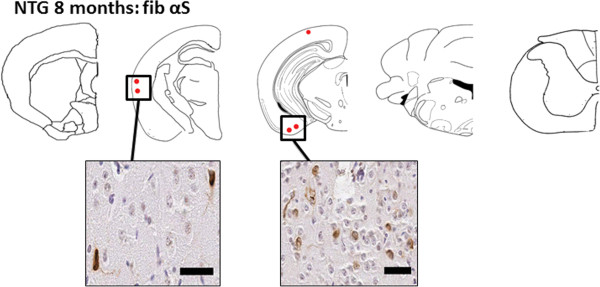
**Schematic summary showing predominant cortical distribution of αS pathology in nTg mice 8 months after brain neonatal injection of fibrillar 21–140 αS.** nTg mice injected with 25 μg 21–140 fibrillar (fib) αS. Map shows rostral-caudal distribution of αS inclusions via coronal sections. Equivalent density and distribution of αS pathology was seen bilaterally. Pathology was detected with antibodies pSer129 and Syn506. As shown in representative images, small, rounded perinuclear αS inclusion and neuritic profiles were found sparsely distributed in the cortex. The distribution of inclusions was very similar in all mice with pathology. Scale bar = 50 μm.

To further investigate induction of αS inclusion formation following brain injection of exogenous αS and to generate a higher throughput experimental model, we injected nTg neonatal mouse brains with exogenous preformed recombinant human αS amyloid fibrils comprised of 21–140 αS (Figure 1). We use amino-truncated 21–140 αS, as fibrils comprised of this protein can seed αS similarly to the full-length protein in cultured cells [38,39,41,42] and it provides the ability to definitively assess aggregation of the endogenous αS by detection with amino-terminal specific αS antibodies. The presence of the exogenous αS (25 μg injected) could be readily detected in the needle track 2 days post injection in nTg mouse brains using an antibody to human αS (Figure 1). By 4 days post-injection, exogenous αS was not detectable, consistent with the findings recently reported by Masuda-Suzukake and colleagues [21] who also showed exogenous human αS injected into the brain was detectable only within the first 7 days post injection. We did not detect local or distal induction of intracellular pathology at 4, 8, and 16 days post-injection of 25 μg fibrillar αS. Analysis of nTg mouse brains neonatally injected with 2 μg of exogenous fibrillar 21–140 αS and aged up to 8 months did not reveal the presence of any αS pathology (Table 1). Injection of 25 μg of exogenous fibrillar 21–140 αS resulted in rare pathology in only one mouse at 2 months post-injection, but at 8 months post-injection, 4 out of 13 nTg mice injected with this dose of αS showed sparse αS neuronal inclusion pathology primarily localized to cortical neurons (Table 1 and Figure 2). In nTg cohorts injected with either 2 μg or 25 μg non-amyloidogenic Δ71-82 αS [34,35,41], we did not observe any pathology in nTg mice (see Table 1). In these studies Δ71-82 αS was used as a control for conformational templating mechanisms, as we and others have extensively studied this protein and showed that it is deficient in the ability to form or directly affect (induce or inhibit) the formation of αS amyloid fibrils *in vitro* or in culture models [34,35,41-43]. After prolonged incubations at high concentrations, Δ71-82 α-synuclein can form oligomers as observed by negative staining electron microscopy, but these are not amyloidogenic in nature. The Δ71-82 α-synuclein used for the current studies was not pre-incubated and is in the soluble form as previously described. We have also recently shown that this same preparation of Δ71-82 α-synuclein cannot directly seed the formation of α-synuclein inclusions in primary neuronal cultures [42]. In contrast the same preparation of fibrillar α-synuclein can seed inclusion formation very efficiently in those cultures.

**Table 1:** **Summary of neonatal nTg mice injected with αS proteins**^**a**^

**Mouse strain**	**Inoculum**	**Age at harvest**	**Number of mice**	**Pathological findings**
C57BL6/C3H	fib αS (2 μl of 1 mg/ml)	1 month	9	No inclusions
C57BL6/C3H	fib αS (2 μl of 1 mg/ml)	2 months	3	No inclusions
C57BL6/C3H	fib αS (2 μl of 1 mg/ml)	4 months	6	No inclusions
C57BL6/C3H	fib αS (2 μl of 1 mg/ml)	8 months	4	No inclusions
C57BL6/C3H	fib αS (5 μl of 5 mg/ml)	1 month	9	No inclusions
C57BL6/C3H	fib αS (5 μl of 5 mg/ml)	2 months	7	1 of 7 mice show rare inclusions^b^
C57BL6/C3H	fib αS (5 μl of 5 mg/ml)	4 months	3	No inclusions
C57BL6/C3H	fib αS (5 μl of 5 mg/ml)	8 months	13	4 of 13 mice show rare cortical inclusions^c^
C57BL6/C3H	Δ71-82 αS (2 μl of 1 mg/ml)	1 month	4	No inclusions
C57BL6/C3H	Δ71-82 αS (2 μl of 1 mg/ml)	2 months	3	No inclusions
C57BL6/C3H	Δ71-82 αS (2 μl of 1 mg/ml)	4 months	2	No inclusions
C57BL6/C3H	Δ71-82 αS (5 μl of 5 mg/ml)	1 month	9	No inclusions
C57BL6/C3H	Δ71-82 αS (5 μl of 5 mg/ml)	2 months	8	No inclusions
C57BL6/C3H	Δ71-82 αS (5 μl of 5 mg/ml)	8 months	6	No inclusions

^a^nTg mice were injected with 21–140 human αS fibrils (fib) or Δ71-82 human αS at the different dosages indicated and analyzed for αS pathology at 1–8 months post-injection using pSer129 and Syn506 antibodies.

^b^Sparse inclusions were observed in the midbrain area of 1 mouse.

^c^ See Figure 2 for a schematic neuroanatomical map showing the distribution of αS pathology.

**Figure 3:**
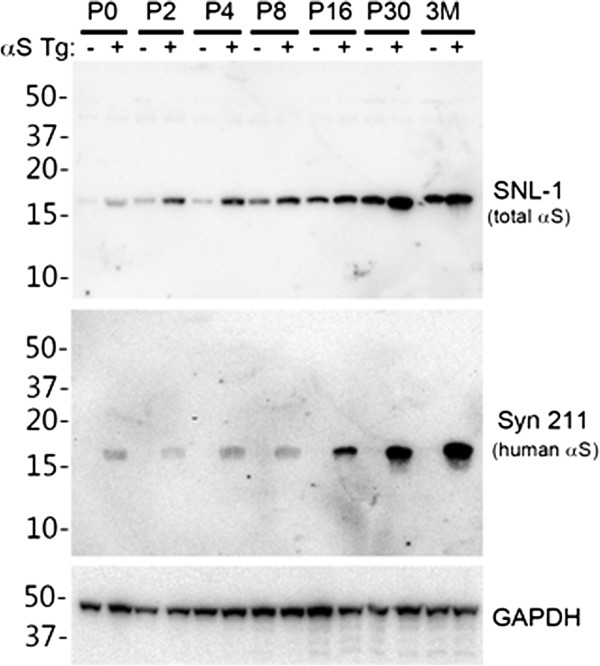
**Increased postnatal expression of αS in the brain of nTg and M20 Tg mice.** Total protein mouse brain extracts from P0, P2, P4, P8, P16, P30, and adult (3 months) nTg (-) and (+) M20 Tg mice was resolved on 13% SDS polyacrylamide gels and analyzed by immunoblotting with anti-αS antibody SNL-1, which detects both human and mouse αS, or anti-human αS antibody Syn211. Immunoblotting with an anti-GAPDH antibody was performed as a loading control. The mobility of molecular mass markers is indicated on the left.

**Figure 4:**
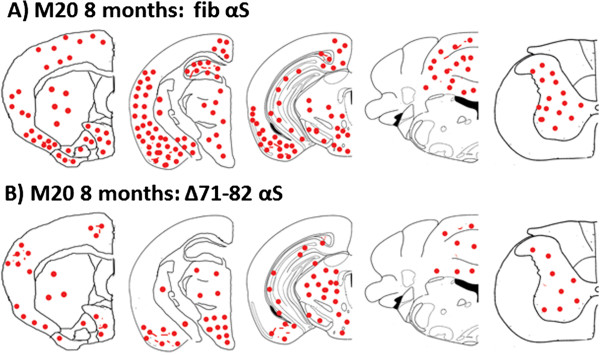
**Schematic representation of the distribution of αS pathology at 8 months following brain neonatal injection of 21–140 human αS fibrils or **Δ**71-82 human in M20 αS Tg mice.** M20 Tg mice injected with 25 μg of fibrillar (fib) 21–140 human αS (**A**) or Δ71-82 human αS (**B**). Maps show rostral-caudal distribution of αS inclusions via coronal sections. Equivalent density and distribution of αS pathology was seen bilaterally. Pathology was detected with antibodies pSer129 and Syn506. (**A**) P0 injection of 21–140 human αS fibrils results in the formation of αS inclusions throughout the cortex, hippocampus, midbrain, brainstem, and spinal cord. (**B**) P0 injection of Δ71-82 human αS also results in widespread αS inclusions. The distribution of inclusions was very similar in all mice with pathology.

**Figure 5:**
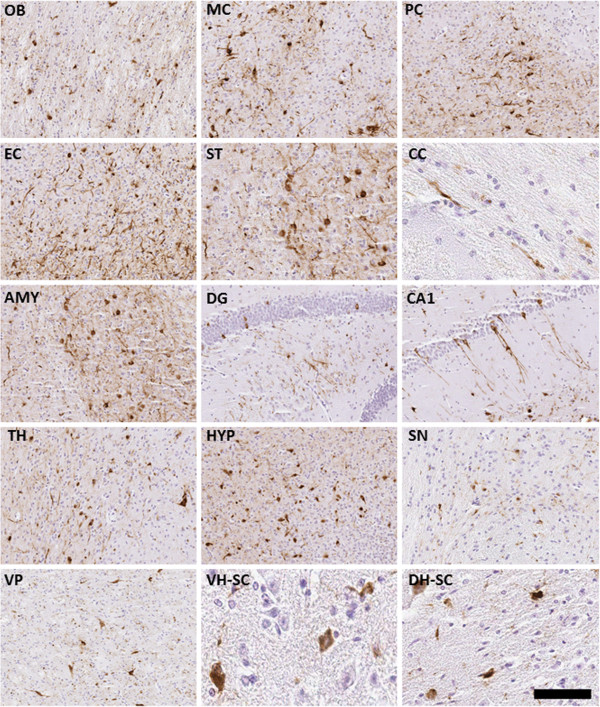
**Induction of αS pathology throughout the neuroaxis 8 months after neonatal brain injection of 25 μg fibrillar 21–140 αS in M20 Tg mice.** Tissue sections were stained with pSer129. Dystrophic neurites were diffusely present throughout the brain and spinal cord. The more rounded, Lewy body-like pathology was seen predominantly in the olfactory bulb (OB), motor cortex (MC), amygdala (AMY), dentate gyrus of the hippocampus (DG), thalamus (TH), hypothalamus (HYP), substantia nigra (SN), ventral pons (VP), and both the ventral and dorsal horns of the spinal cord (VH-SC and DH-SC). Lewy neurite-like pathology extending into the cellular processes was more predominantly seen in the piriform cortex (PC), entorhinal cortex (EC), striatum (ST), corpus callosum (CC), and CA1 of the hippocampus (CA1). Tissue sections were counterstained with hematoxylin. Scale bar = 50 μm (OB), 100 μm (MC), 50 μm (PC), 50 μm (EC), 100 μm (ST), 200 μm (CC), 50 μm (AMY), 50 μm (DG), 50 μm (CA1), 50 μm (TH), 50 μm (HYP), 50 μm (SN), 50 μm (VP), 200 μm (VH-SC), and 200 μm (DH-SC).

**Figure 6:**
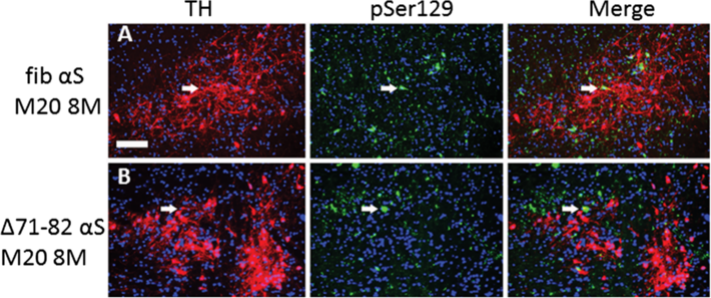
**The majority of αS inclusions in the substantia nigra of M20 Tg mice neonatally injected with exogenous αS are not in TH positive neurons.** M20 Tg mice 8 months after neonatal injection with 21–140 human αS fibrils (fib) (**A**) or Δ71-82 human αS (**B**). Double-labeled immunofluorescence analysis for tyrosine hydroxylase (TH; red) labeling the dopaminergic neurons in the substantia nigra area, and pSer129 (green) labeling the hyperphosphorylated αS inclusions show minimal co-localization. The majority of αS inclusions were not found in TH+ cells, except for a few neurons (white arrows). Cell nuclei were counter stained with DAPI. Scale bar = 100 μm.

**Figure 7:**
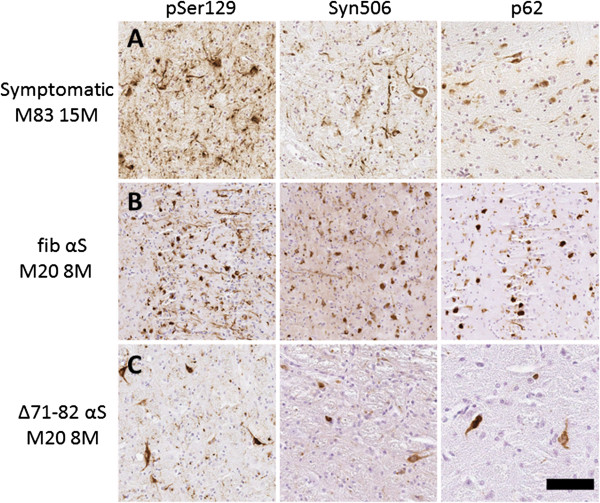
**IHC showing similar αS pathology induced by neonatal brain injection of fibrillar human 21–140 αS and **Δ**71-82 human αS in M20 Tg mice compared to a symptomatic M83 Tg mouse.** Brainstem tissue sections from a 15 month-old symptomatic M83 Tg mouse (**A**) and 8 month-old M20 Tg mice neonatally injected in the brain with 25 μg 21–140 fibrillar (fib) αS (**B**) or 25 μg Δ71-82 (**C**) show similar staining of αS inclusions as detected with pSer129 by IHC. αS inclusions are also detected with Syn506 and p62 antibodies. Syn506 is a mouse monoclonal antibody that conformationally detects αS inclusions; and p62 is a rabbit polyclonal antibody, which non-specifically recognizes intracellular protein aggregates. Scale bar = 100 μm.

**Figure 8:**
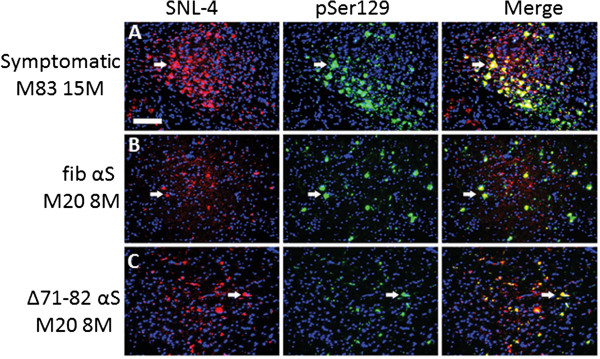
**Detection of αS inclusions in M20 Tg mice 8 months after PO brain injection of exogenous αS with both amino-terminal αS antibody SNL-4 and pSer129.** Double-labeled immunofluorescence of midbrain with SNL-4 (red) and pSer129 (green) shows that pSer129+ hyperphosphorylated αS inclusions are SNL-4+ in a symptomatic 15 month-old M83 Tg mouse (**A**) and 8 month-old M20 Tg mice neonatally injected in the brain with 25 μg 21–140 fibrillar (fib) αS (**B**) or 25 μg Δ71-82 (**C**). Cell nuclei were counter stained with DAPI. Scale bar = 100 μm.

**Figure 9:**
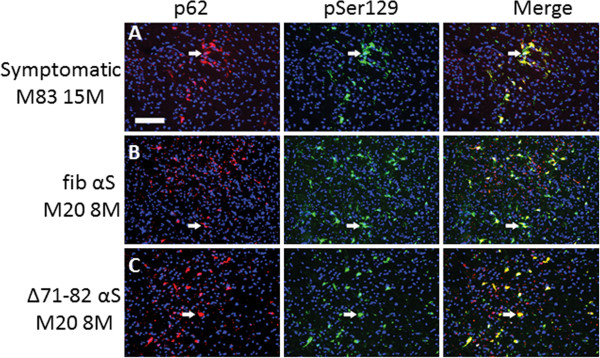
**Co-localization of p62 with αS inclusions in 8 month-old M20 Tg mice following neonatal brain injection of exogenous αS.** Double-labeled immunofluorescence analysis in the midbrain region for p62 (red) and pSer129 (green) showing that most pSer129+ hyperphosphorylated αS inclusions are p62+ in a symptomatic 15 month-old M83 Tg mouse (**A**) and 8 month-old M20 Tg mice neonatally injected in the brain with 25 μg 21–140 fibrillar (fib) αS (**B**) or 25 μg Δ71-82 (**C**). Cell nuclei were counter stained with DAPI. Scale bar = 100 μm.

**Figure 10:**
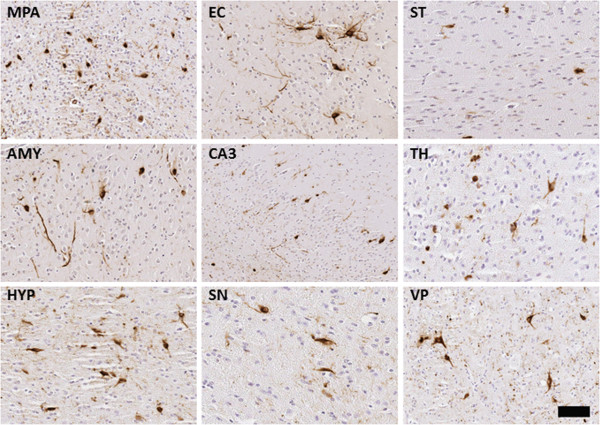
**Induction of αS pathology throughout the neuroaxis 8 months after neonatal brain injection of 25 μg **Δ**71-82 αS in M20 Tg mice.** Tissue sections were stained with pSer129. Round perikaryal inclusions and dystrophic neurites were diffusely spread throughout the brain and spinal cord. The more rounded, Lewy body-like pathology was seen predominantly in the medial preoptic area (MPA), striatum (ST), thalamus (TH), hypothalamus (HYP), substantia nigra (SN), and ventral pons (VP). Lewy neurite-like pathology extending into the cellular processes was more predominantly seen in the entorhinal cortex (EC), amygdala (AMY), and CA3 region of the hippocampus (CA3). Tissue sections were counterstained with hematoxylin. Scale bars = 100 μm (MPA), 50 μm (EC), 100 μm (ST), 100 μm (AMY), 200 μm (CA3), 50 μm (TH), 100 μm (HYP), 50 μm (SN), and 100 μm (VP).

To examine whether overexpression of αS can increase the efficiency of inclusion pathology formation *in vivo*, we performed neonatal brain injection of fibrillar 21–140 αS in M20 Tg mice, which overexpress wild type human αS. In adult M20 Tg mice there is ~5-fold over expression of human αS in the brain, but these mice do not develop αS pathology during their lifespan in the absence of additional manipulations [31,32]. These mice also overexpress transgenic human αS during development that can be observed as early as P0 (Figure 3), and as previously reported, the expression of αS increases during mouse brain development [44]. Thus, they make an ideal model to explore paradigms for induction of αS pathology. The neonatal brain injection of 2 μg fibrillar 21–140 αS did not induce the formation of intraneuronal pathology at times up to 4 months, but by 8 months sparse cortical pathology could be observed (Table 2). Similar challenge to Δ71-82 αS did not result in the formation of pathology. Increasing the treatment to 25 μg fibrillar 21–140 αS resulted in sparse brain αS pathology as early as 1 month and 4 months, but it was extensively distributed throughout the neuroaxis by 8 months (Table 2, Figures 4 and 5) at a higher density than in nTg mice showing αS pathology at 8 months post-injection of 25 μg fibrillar 21–140 αS. Interestingly, αS inclusions were rarely observed in nigral dopaminergic neurons (Figure 6). Similar to the αS aggregates in symptomatic M83 Tg mice, which spontaneously develop age-dependent pathology [31,32], the inclusions in M20 Tg mice induced by the brain injection of fibrillar 21–140 αS were comprised of endogenously expressed αS as they were reactive with amino-terminal specific antibodies Syn506 and SNL-4 (Figures 7 and 8). The inclusions were also reactive with p62 (sequestrosome; Figure 9), a robust marker of αS inclusions [45]. Unexpectedly, similar brain injection of Δ71-82 αS also resulted in robust and widely distributed αS brain pathology at 8 months in some of the injected M20 Tg mice (Table 2; Figures 6, 7, 8, 9 and 10). These αS inclusions were also comprised of endogenous αS (i.e. reactive with antibodies Syn506 and SNL-4) hyperphosphorylated at Ser129, and accumulated p62. For comparison, we show that some of the M20 Tg mice 8 months post-injection with 25 μg of Δ71-82 αS are devoid of αS pathology (see Additional file 1: Figure S1). Although none of the nTg or the M20 Tg mice were extensively analyzed for behavioral changes, the presence of αS pathological inclusions was not associated with any overt behavioral abnormalities.

**Table 2:** **Summary of neonatal M20 Tg mice injected with αS proteins**^**a**^

**Mouse strain**	**Inoculums**	**Age at harvest**	**Number of mice**	**Pathological findings**
M20 (WT αS)	fib αS (2 μl of 1 mg/ml)	1 month	7	No inclusions
M20 (WT αS)	fib αS (2 μl of 1 mg/ml)	2 months	5	No inclusions
M20 (WT αS)	fib αS (2 μl of 1 mg/ml)	4 months	3	No inclusions
M20 (WT αS)	fib αS (2 μl of 1 mg/ml)	8 months	4	4 of 4 mice show sparse cortical pathology
M20 (WT αS)	fib αS (5 μl of 5 mg/ml)	1 month	4	3 of 4 mice show sparse cortical pathology
M20 (WT αS)	fib αS (5 μl of 5 mg/ml)	2 months	5	4 of 5 mice show sparse cortical pathology
M20 (WT αS)	fib αS (5 μl of 5 mg/ml)	4 months	3	2 of 3 mice show sparse cortical pathology
M20 (WT αS)	fib αS (5 μl of 5 mg/ml)	8 months	12	12 of 12 mice show abundant pathology^b^
M20 (WT αS)	Δ71-82 αS (2 μl of 1 mg/ml)	1 month	5	No inclusions
M20 (WT αS)	Δ71-82 αS (2 μl of 1 mg/ml)	2 months	5	No inclusions
M20 (WT αS)	Δ71-82 αS (2 μl of 1 mg/ml)	4 months	3	No inclusions
M20 (WT αS)	Δ71-82 αS (2 μl of 1 mg/ml)	8 months	3	No inclusions
M20 (WT αS)	Δ71-82 αS (5 μl of 5 mg/ml)	1 month	7	No inclusions
M20 (WT αS)	Δ71-82 αS (5 μl of 5 mg/ml)	2 months	7	No inclusions
M20 (WT αS)	Δ71-82 αS (5 μl of 5 mg/ml)	8 months	6	2 out of 6 mice show abundant pathology^b^

^a^ M20 Tg mice were injected with 21–140 human αS fibrils (fib) or Δ71-82 human αS at the different dosages indicated and analyzed for αS pathology at 1–8 months post-injection using pSer129 and Syn506 antibodies.

^b^ See Figure 4 for a schematic neuroanatomical map showing the distribution of αS pathology.

**Figure 11:**
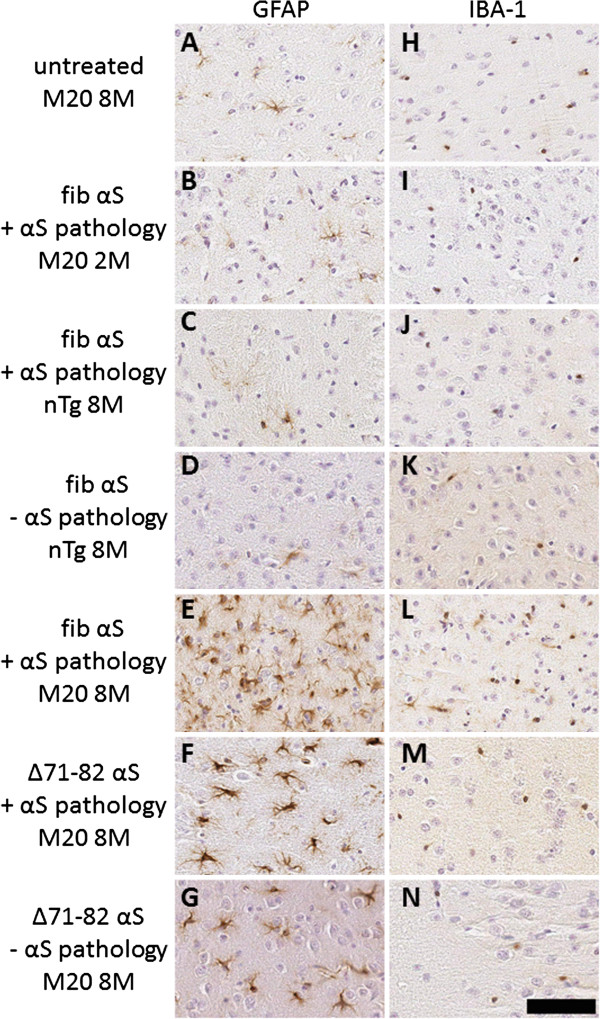
**Delayed induction of astrogliosis and microgliosis in mice neonatally injected with soluble **Δ**71-82 αS or fibrillar αS at 8 months post-injection.** Tissue sections were stained with GFAP antibody (**A**-**G**), which detects astrocytes, and IBA-1 antibody (**H-N**), which detects microglia. Representative images were taken of the entorhinal cortex, where a high density of αS pathology tends to form due to neonatal injection (see Figures 2, 4, 5 and 10). An 8-month-old control untreated M20 Tg mouse (**A**, **H**) and a M20 Tg mouse injected with 25 μg of 21–140 fibrillar (fib) αS at 2 months post-injection (**B**, **I**) show basal levels of astrocytes and microglia. There was no significant increase from basal levels in the brains of a nTg mouse with pathology (**C**,** J**) relative to a similar nTg mouse without pathology (**D**, **K**) at 8 months post-injection of 25 μg of 21–140 fibrillar αS. Robust astrocyte and microglia activation as observed 8 months after injection in M20 Tg mice with brain αS pathology treated with 25 μg of 21–140 fibrillar αS (**E**, **L**). In addition, robust astrogliosis was also observed in M20 Tg mice 8 months after injection of 25 μg of Δ71-82 αS with (**F**, **M**), or without (**G**, **N**) brain αS pathology. Tissue sections were counterstained with hematoxylin. Scale bar = 50 μm.

To assess if there was an association between neuroinflammation (astrogliosis or microgliosis) and induction of αS aggregation, tissue sections from all injected nTg and M20 Tg mice were stained with antibodies to GFAP and IBA-1. As expected, control untreated M20 Tg mice at 8 months of age showed basal levels of astrocytes and microglia (Figure 11A and 11H) [17,32]. Most injected nTg and M20 Tg mice at 1, 2 or 4 month post-injection did not display increased astrogliosis or microgliosis as shown for M20 Tg mice 2 months post-injection with 25 μg fibrillar αS (Figure 11B and 11I). At these ages some of the M20 Tg mice with or without brain αS pathology also revealed a modest increase in astrogliosis. In 8 month old nTg mice injected with fibrillar αS and with modest αS pathology, only minimal induction of astrocytes and microglia was observed (Figure 11C, 11D, 11J and 11K). Conversely, in 8 month old M20 Tg mice with significant αS pathology induced by neonatal injection of 25 μg fibrillar αS, robust astrogliosis and modest microgliosis were observed (Figure 11E, 11L). Furthermore, in 8 month old M20 Tg mice injected with 25 μg Δ71-82 αS, there was also robust astrogliosis (Figure 11F, 11G, 11M, and 11N) regardless of whether αS pathology had developed. These findings indicate that treatment with fibrillar or non-amyloidogenic αS can induce a delayed activation of neuroinflammation that is significantly accentuated in M20 Tg mice relative to nTg mice.

**Figure 12:**
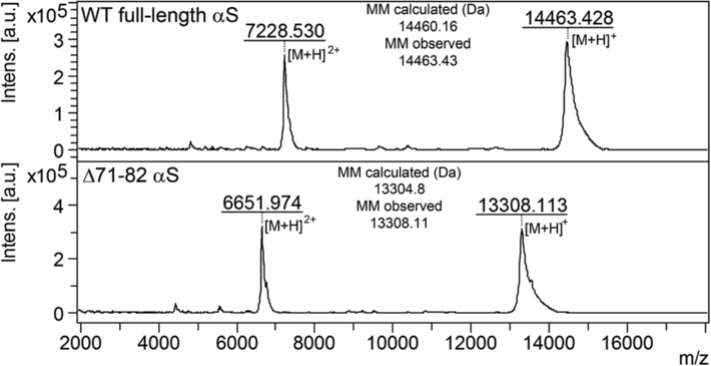
**Mass spectrometric analysis of the **Δ**71-82 αS used for neonatal brain injection.** To verify that Δ71-82 αS was the correct protein and its integrity, we performed mass spectrometry and compared the molecular mass to full length αS. Up panel, recombinant full-length human αS wild type; bottom panel, recombinant human Δ71-82 αS.

To evaluate the purity, biophysical properties, and integrity of the recombinant Δ71-82 αS, we performed MS analysis (Figure 12). We confirmed by K114 fluorometry that Δ71-82 αS protein was not amyloidogenic as previously described [35], and that the addition of exogenous Δ71-82 αS could not induce αS inclusion formation in cultured cells [42].

## Discussion and conclusion

Our studies demonstrate that the brain injection of exogenous αS can induce intraneuronal αS pathology after prolonged incubation times. Within days the injected αS is rapidly cleared and the inclusion pathology that arises from endogenously expressed αS takes months to form. These findings are consistent with those of Masuda-Suzukake and colleagues who showed that exogenously injected human αS fibrils (10 μg) into the brains of nTg mice can be detected for less than 1 week, but induction of αS pathology is observed 3 months later [21]. All prior studies of intraneuronal induction of αS by cerebral challenge to exogenous fibrils have been interpreted as being indicative of a “prion-like” spread of αS pathology [18,20,21]. Indeed the delayed induction of αS pathology by exogenous αS observed here and by Masuda-Suzukake et al. [21] may be interpreted as stable αS seeds that are present below detectable levels. Over time these seeds induce pathology, which then may spread via a cycle of inclusion pathology giving rise to additional nucleation events that can be spread from cell to cell. Our findings that an injection of a non-amyloidogenic form of αS (Δ71-82) can induce similar delayed pathology indicate that it may be premature to conclude that the pathology induced is solely attributable to conformational dependent templating events. In both the Masuda-Suzukake et al. [21] and Luk et al. [20] studies using nTg mice, soluble αS was injected as controls and no induction of pathology was reported. As we find that at a higher dose of αS there is more robust induction of αS pathology both in terms of extent of pathology and time to onset of pathology induction, and that injection of αS in M20 Tg mice also enhances the resultant inclusion pathology phenotype, it is possible that the lower doses (5–10 μg) of injected soluble αS in those studies [20,21] may account for the lack of pathology induction reported. Notably, a soluble αS control injection was not reported in the study demonstrating pathology induction in adult M83 Tg mice [18]. Here using neonatal αS Tg mice, we observed that injection of soluble Δ71-82 αS is capable of inducing αS pathology similar to amyloidogenic αS. Induction of robust intraneuronal αS pathology by exogenous Δ71-82 αS challenge does not appear to be attributable to the neonatal injection paradigm as we have observed similar findings in adult mice (Sacino et al., in preparation). The finding that M20 Tg mice are more prone to inclusion formation resulting from treatment with either exogenous fibrillar αS or soluble Δ71-82 αS is likely due to a dosage effect of αS expression, which could be akin to patients with duplication or triplication of the *SNCA* gene. Although the cohorts of mice used here are not large, they are comparable to those used by others to study the induction of brain pathology using injected exogenous αS [18,20,21]. Larger cohorts of mice injected with various dosages of αS are currently being aged to longer time points to further understand the mechanisms involved in exogenous αS induction of brain pathology.

Circumstantial evidence from post-mortem studies of the distribution of αS pathology in the brains of PD patients as well as the induction of αS pathology in transplanted neurons in the brains of some PD patients has been used to support the hypothesis that αS pathology may spread from cell-to-cell by a “prion-like” mechanism [13,14,46-48]. However, many alternative explanations including chronic neuroinflammation, oxidative stress triggered by excitotoxicity, and loss of homeostasis from cellular stress, may lead to the failure of molecular chaperones and other machinery to effectively control the level of misfolded αS [22-25].

Our data that soluble non-amyloidogenic αS can induce widespread αS pathology raises questions regarding the “prion-like” spread of pathology that has been reported, but it is premature to conclude that our studies definitively refute that mechanism. It is plausible that in the brain a non-amyloidogenic αS could be converted into amyloidogenic seeds through additional modifications or interactions with lipids or protein chaperones. Thus, studies that track the fate of injected exogenous αS will be necessary to evaluate these possibilities.

Alternatively, these studies do strongly suggest that other mechanism(s) of induction of pathology by exogenous αS should be considered. For example, there are extensive reports on how extracellular αS can lead to activation of the innate immune response via toll-like receptor pathways akin to lipopolysaccharide activation [49-62], and single intracerebellar or intraperitoneal injections of lipopolysaccharide have been shown to result in the long-lasting induction of αS neuronal inclusion formation [63,64]. Importantly, αS lacking residues 71–82 has been shown to induce inflammation similar to full-length αS [54,59]. In our current study, we have observed a delayed, long-term activation of neuroin flammation induced by brain treatment to both soluble and fibrillar αS that is accentuated in M20 Tg mice compared to nTg mice. These findings are consistent with cell culture studies that showed that both soluble and aggregated αS are potent activators of inflammation [49-62]. It is possible that the exogenous treatment of αS may trigger a slow positive feedback loop of inflammation and secretion followed by aggregation that may require a certain threshold of inflammation that builds overtime. Therefore, some of the M20 Tg mice with neuroinflammation, but without αS inclusions 8 months after treatment with Δ71-82 αS may not yet have reached the necessary threshold. Exogenous Δ71-82 αS may not be as potent an inducer of this process as is fibrillar αS, because it may have a shorter half-life than aggregated αS, which could explain why it was not as potent as fibrillar αS; however, this possibility will be investigated in future studies. However, the hypothesis that inflammation may play an important role in the spread of αS pathology induced by exogenous αS is only one of several possible mechanisms that may act synergistically or independently to promote the spread of αS pathology [22-25].

Furthermore, there is abundant evidence that prionoid self-protein aggregates represent what are referred to immunologically as Danger Associated Molecular Patterns (DAMPs) and are capable of inducing robust immune responses [65]. A number of studies show that when prionoids associated with CNS proteinopathies are applied exogenously to glial cells they can activate innate immunity through pattern recognition receptors (PRR) and induce a proinflammatory response [65-68]. This innate immune response in turn could trigger inclusion pathology. Notably, these mechanisms are not mutually exclusive and may be mutually self-reinforcing [25]. Our impression from these studies is that amyloidogenic αS is a more efficient inducer of pathology than the non-amyloidogenic αS. This differential efficiency could be attributed to a myriad of different properties ranging from stability of the aggregated αS, differential ability to template pathology, immunogenicity, or some combination of these factors.

Our recent studies in cultured cell provide strong evidence that amyloidogenic conformational templating of αS can readily occur under certain conditions [42], but the situation *in vivo* is likely more complex and aggregate formation can involve several mechanisms. To this point, treatment with Δ71-82 αS did not induce αS aggregation in cultured cells, while fibrillar αS was able to readily do so. A limitation of studies in culture cells is the duration of time (a few weeks) that the cells can be maintained experimentally, which may not be efficient to study mechanisms that are slower and more progressive. Collectively these data indicate that exposure to exogenous αS can induce intracellular aggregate formation by at least 2 mechanisms that are not mutually exclusive and could likely be synergistic.

Further studies will be needed to determine the relative contribution of “prion-like” protein self-templating versus other mechanisms in the induction and propagation of αS pathology. Notably, the neonatal injection paradigm that we have developed can accelerate these mechanistic studies by reducing the time needed to establish cohorts of mice necessary to conduct those studies. As non-amyloidogenic αS can induce αS pathology similar to fibrillar amyloidogenic αS, it is possible that any form of brain injury that promotes release of normal cellular αS could trigger intraneuronal αS pathology. Extracellular αS release could also occur during neurodegeneration when neurons die and this could be exacerbated if the protein is not cleared rapidly. More definitive elucidation of the mechanism(s) that underlie induction and spread of αS pathology are likely to provide key insights into ongoing efforts designed to target αS *in vivo* and thereby ultimately lead to novel disease modifying therapies for PD and other α-synucleinopathies.

## Competing interest

The authors declare that they have no conflict of interest.

## Authors’ contributions

ANS designed the study, performed the injections, analyzed the data, and drafted the manuscript. MB collected the samples and analyzed the data. NHM analyzed the data. ABM performed the injections and analyzed the data. MAT performed the injections and analyzed the data. YL participated in coordination of study. YR participated in the design of the study and performed the mass spectrometric analysis. TEG and BIG participated in the design and coordination of the study, analyzed the data, and helped to draft the manuscript. All authors read and approved the final manuscript.

## Supplementary Material

Additional file 1: Figure S1 **Lack of induction of αS pathology throughout the neuroaxis 8 months after neonatal brain injection of 25 μg **Δ**71-82 αS in M20 Tg mice.** Tissue sections were stained with pSer129. Brain regions that typically showed Lewy body/neurite-like pathology after injection of 25 μg Δ71-82 αS in M20 Tg mice, were blank in an unaffected mouse: the medial preoptic area (MPA), striatum (ST), thalamus (TH), hypothalamus (HYP), substantia nigra (SN), ventral pons (VP), entorhinal cortex (EC), amygdala (AMY), and CA3 region of the hippocampus (CA3). Tissue sections were counterstained with hematoxylin. Scale bars = 100 μm (MPA), 50 μm (EC), 100 μm (ST), 100 μm (AMY), 200 μm (CA3), 50 μm (TH), 100 μm (HYP), 50 μm (SN), and 100 μm (VP). Click here for file
