# Supplementary figures and images for: Induction of CNS α-synuclein pathology by fibrillar and non-amyloidogenic recombinant α-synuclein
Source: Acta Neuropathol Commun. 2013 Jul 17;1:38. doi: 10.1186/2051-5960-1-38 (PMC3893388; doi:10.1186/2051-5960-1-38)

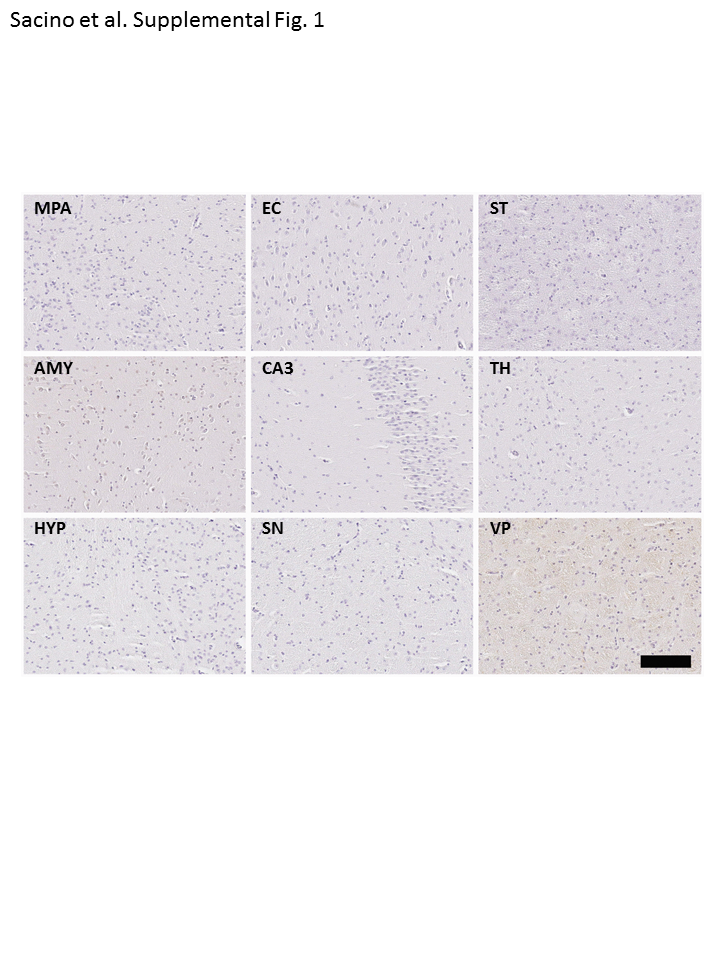

Supplement: Additional file 1: Figure S1 — Lack of induction of αS pathology throughout the neuroaxis 8 months after neonatal brain injection of 25 μg Δ71-82 αS in M20 Tg mice. Tissue sections were stained with pSer129. Brain regions that typically showed Lewy body/neurite-like pathology after injection of 25 μg Δ71-82 αS in M20 Tg mice, were blank in an unaffected mouse: the medial preoptic area (MPA), striatum (ST), thalamus (TH), hypothalamus (HYP), substantia nigra (SN), ventral pons (VP), entorhinal cortex (EC), amygdala (AMY), and CA3 region of the hippocampus (CA3). Tissue sections were counterstained with hematoxylin. Scale bars = 100 μm (MPA), 50 μm (EC), 100 μm (ST), 100 μm (AMY), 200 μm (CA3), 50 μm (TH), 100 μm (HYP), 50 μm (SN), and 100 μm (VP). [file 2051-5960-1-38-S1.tiff]
